# Heart Rate Responses during Sport-Specific High-Intensity Circuit Exercise in Child Female Gymnasts

**DOI:** 10.3390/sports8050068

**Published:** 2020-05-18

**Authors:** Andreas Salagas, Olyvia Donti, Christos Katsikas, Gregory C. Bogdanis

**Affiliations:** School of Physical Education and Sports Science, National and Kapodistrian University of Athens, 172 37 Athens, Greece; andreassal@phed.uoa.gr (A.S.); odonti@phed.uoa.gr (O.D.); ckatsikas@phed.uoa.gr (C.K.)

**Keywords:** aerobic fitness, intermittent exercise, prepubertal children

## Abstract

This study examined heart rate (HR) responses during a sport-specific high-intensity circuit training session to indirectly assess cardiorespiratory stress in child athletes. Seventeen, female gymnasts, aged 9–11 years performed two 5-min 15 s sets of circuit exercise, interspersed by a 3 min rest interval. Each set included five rounds of five gymnastic exercises (7 s work, 7 s rest) executed with maximal effort. During the first circuit training set, peak heart rate (HR) was 192 ± 7 bpm and average HR was 83 ± 4% of maximum HR (HR_max_), which was determined in a separate session. In the second set, peak HR and average HR were increased to 196 ± 8 bpm (*p* < 0.001, *d* = 0.55) and to 89 ± 4% HR_max_ (*p* < 0.001, *d* = 2.19), respectively, compared with the first set. HR was above 80% HR_max_ for 4.1 ± 1.2 min during set 1 and this was increased to 5.1 ± 0.4 min in set 2 (*p* < 0.001, *d* = 1.15). Likewise, HR was above 90% of HR_max_ for 2.0 ± 1.2 min in set 1 and was increased to 3.4 ± 1.7 min in set 2 (*p* < 0.001, *d* = 0.98). In summary, two 5-min 15 s sets of high-intensity circuit training using sport-specific exercises, increased HR to levels above 80% and 90% HR_max_ for extended time periods, and thus may be considered as an appropriate stimulus, in terms of intensity, for improving aerobic fitness in child female gymnasts.

## 1. Introduction

A large number of studies over the last decade have shown that high-intensity interval training (HΙΙΤ) improves athletic performance and health in adults [1,2,3]. HIIT typically includes short duration exercise bouts (15–60 s) performed at an intensity around maximal oxygen uptake (VO_2max_) [4,5], or shorter bouts (6–15 s) executed at intensities corresponding to 100–130% of VO_2max_ with work-to-rest ratios of 1:1 to 1:1.5 [4,6]. Previous studies have shown significant aerobic contribution during high-intensity exercise in adults [7], while children demonstrate even higher reliance on their aerobic metabolism in this type of exercise, due to their faster VO_2_ kinetics and lower glycolytic energy supply [8,9]. However, the majority of HIIT studies in children used intense running or cycling [5,8,10,11], and little is known regarding the aerobic contribution during other forms of high-intensity exercise, such as functional training, with most data obtained from adult populations [12,13]. This type of training is commonly used by coaches in many sports, such as gymnastics, and typically includes sport-specific exercises using body weight, which are executed in a circuit fashion, aiming to improve neuromuscular performance [14]. However, there is very limited information about the acute cardiorespiratory stress for this type of circuit training program, especially in child athletes who are systematically training from a very young age [15].

Artistic gymnastics is a popular sport that requires high levels of strength, power, flexibility, coordination and anaerobic power [14]. During training sessions, gymnastic routines and exercises are performed repetitively over a long period of time, with short recovery intervals. Thus, the ability to recover is important not only to preserve a high quality of technical execution throughout a training session, but also for optimal performance during competitions [14]. Previous studies have shown that aerobic fitness is an important determinant of performance recovery, while the aerobic contribution to energy supply is substantial when high-intensity efforts last 20–30 s or longer [7,16]. For example, in artistic gymnastics, VO_2_ during competitive routines of floor exercises lasting 90 s, is increased to 85% of maximal VO_2_ (VO_2max_) [17]. Similarly, peak heart rate reaches values over 90% of maximum heart rate in all apparatuses except the vault, where heart rate does not increase to such high levels, due to the short duration of a single vault (5–7 s) [17]. Furthermore, VO_2max_ explained 92.5% of the variation in performance scores in elite rhythmic gymnasts, whose competitive routines last about 60–90 s [18]. Thus, aerobic fitness may be important for artistic gymnasts’ performance, and although high-intensity circuit training using sport-specific exercises is used by gymnastics coaches from an early age [14,19], evidence is limited regarding the physiological stress imposed on the cardio-respiratory system of developing athletes. Thus, the aim of this study was to examine heart rate responses during a high-intensity circuit training session using sport-specific exercises in child female gymnasts.

## 2. Materials and Methods

### 2.1. Participants

Participants were recruited from a local gymnastics club. The inclusion criteria were: (1) healthy female athletes, (2) participation in competitive artistic gymnastics for 3–4 years, (3) weekly training for at least 4 hours, and (4) age range between 9–11 years. Athletes who had any musculoskeletal injury from the previous 6 months were excluded from the study. Seventeen premenarcheal female artistic gymnasts aged 9.7 ± 0.8 years, with body mass 33.7 ± 7.3 kg, height 1.38 ± 0.10 cm and Body Mass Index 17.4 ± 2.4 kg/m^2^, participated in the testing procedures. All procedures were in accordance with the Declaration of Helsinki and approved by the local university ethics committee (approval no. 1198). Parents and participants were informed about the experimental procedure and signed an informed consent. All participants had an athlete’s health card validated by the Hellenic Gymnastics Federation.

### 2.2. Procedure

The experimental protocol was performed in the pre-season. Following two familiarization sessions performed 2–3 days apart, the participants executed a 20 m shuttle run test until exhaustion, to measure maximal heart rate (HR_max_) and to estimate maximal oxygen consumption (VO_2max_), using a standardized age-specific equation [20]. Three days after the shuttle run test, the main testing protocol was performed, which consisted of two sets of five gymnastic exercises executed in a circuit manner (Table 1). Participants abstained from any rigorous physical activity for 24 h before testing. Each exercise was performed for 7 s, followed by a rest interval of equal duration, during which athletes moved to the next exercise (Table 1). These five exercises were executed in a circuit fashion until a total of 5 rounds was completed. Thus, each set included 5 rounds of 5 exercises with a total duration of 5 min and 15 s. A passive recovery period of 3 min separated the two sets. A 5 min standardized, sport-specific warm-up preceded the circuit training session, followed by 3 min of rest. The warm-up included 3 min of light jogging and 2 min of mobility exercises.

### 2.3. Heart Rate Measurements

**Figure 1:**
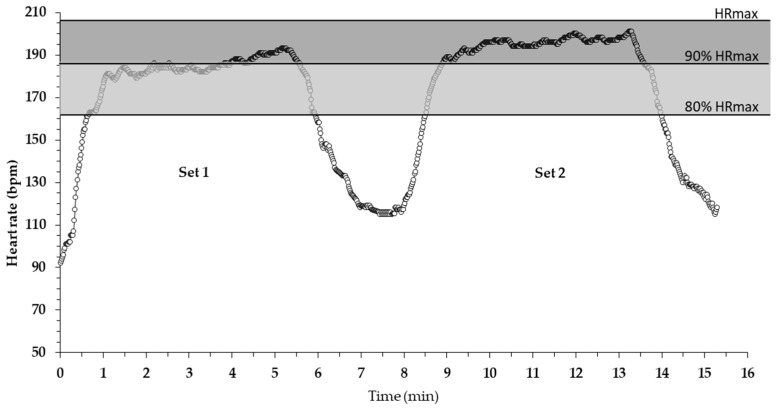
Time course of heart rate during the two sets of circuit training, separated by a 3 min rest interval, for one of the participants.

During the 20 m shuttle run test, subjects’ heart rate was monitored continuously using online telemetry (Polar Team 2, Polar Electro Oy, Kempele, Finland). During the main testing procedure, heart rate (HR) was being measured continuously (every 1 s) for the entire duration of the protocol, i.e., during set 1 and set 2, including 3 min of recovery after each set (see Figure 1). From the heart rate data, the following parameters were extracted or calculated: (a) peak HR, (b) mean HR, (c) time during which heart rate was above 80% of HR_max_, (d) time during which heart rate was above 90% of HR_max_, (e) heart rate recovery 1 and 2 min after each set of the circuit training (i.e., the drop of HR at the respective time points compared with the peak attained in each set) [21,22].

### 2.4. Statistical Analysis

Data analysis was performed using SPSS Statistics (Ver. 25, IBM Corporation, New York, NY, USA). Descriptive statistics were calculated (mean values and standard deviations). Comparisons between the heart rate variables of the first and the second test were performed using a paired-sample T-test. Effect sizes were determined by Cohen’s d (trivial: 0–0.19, small: 0.20–0.49, medium: 0.50–0.79 and large: 0.80 and greater) [23]. One-way analysis of variance (ANOVA) followed by Tukey’s post-hoc test, was used to examine whether peak heart rate and heart rate recovery observed during the two sets of circuit exercise session were different from the respective values (i.e., maximal heart rate and heart rate recovery) recorded during the shuttle run test. Significance was accepted at *p* < 0.05.

## 3. Results

### 3.1. Circuit Exercise Session

Peak heart rate reached in set 1 of the circuit exercise training session was 92 ± 4% of HR_max_, and was increased to 95 ± 4% of HR_max_ in set 2 (Table 2). Mean heart rate during the first set of the circuit exercise session was 83 ± 4% HR_max_ and was further increased to 89 ± 4% HR_max_ in set 2 (Table 2). Moreover, the time during which HR was above 80% HR_max_ and 90% HR_max_ was higher in set 2 compared with set 1 (*p* < 0.00, *d* = 1.15) (*p* < 0.001, *d* = 0.98) (Table 2). The time course of heart rate during the high-intensity circuit training session from a representative individual is shown in Figure 1.

### 3.2. Shuttle Run Test

HR_max_ attained during the shuttle-run test was 207 ± 5 beats per minute (bpm), while the estimated VO_2max_ was 49 ± 3 mL/kg/min HR_max_ attained during the shuttle run test to exhaustion (*p* < 0.001) was 5–8% higher compared with the peak HR during set 1 and set 2 of the circuit training exercise session. However, the recovery of HR after the shuttle run test was similar to HR recovery observed in set 1 and set 2 of the circuit exercise training session (1st min: 58 ± 16 and 2nd min: 76 ± 13 bpm, *p* = 0.26 to 0.65, Table 2).

## 4. Discussion

The main finding of this study was that this sport-specific high-intensity circuit training, which comprised a total exercise time of 10.5 min, increased mean HR to levels above 80% HR_max_ for a total of 9.2 min, and above 90% HR_max_ for a total of 5.4 min (sum of time in set 1 and set 2, see Table 2). This extended time spent at a high HR may be an appropriate stimulus for improvements in aerobic fitness in very young female gymnasts. These findings are important, since circuit training using functional sport-specific exercises is routinely used in developing athletes, mainly to improve neuromuscular fitness [24]. However, exercise performed intermittently at a high intensity has been shown to improve not only strength and muscle endurance, but also to involve a significant aerobic contribution [25,26].

One interesting observation is that the time during which the heart rate was >80% HR_max_ was about 70% of the exercise plus recovery duration. Notably, these young athletes spent 9.2 min out of a total of ~16 min of exercise and recovery (2 × 5:15 min separated by 3 min or rest) with a high HR (Table 2). Furthermore, during 5.4 min of this time, gymnasts had an HR above 90% of HR_max_ (Figure 1). This time spent at a high HR is an adequate stimulus for improving VO_2max_ and aerobic fitness in general [4,27]. The fact that such a large part of this brief exercise scheme was performed with a high HR may be due to the rapid HR kinetics of children, together with their higher oxidative capacity and aerobic contribution to high-intensity exercise [8,28]. Thus, these findings provide evidence that cardiorespiratory stress is high during this type of high-intensity, sport-specific circuit training. Interestingly, a very recent study compared the acute effects of an integrative neuromuscular training program for 12 min (2 sets × 6 exercises × 30 s each with equal rest) on cardiometabolic responses of 10–11 year old children [15]. In that study, the increase of HR was lower than in the present study and HR ranged between 61% and 92% of HR_max_ [15]. Importantly, in that study, VO_2_ during the 12 min exercise was increased from 28% to 64% VO_2max_, suggesting that HR is indicative of oxygen uptake in this type of protocol. This would suggest that in the present study, more than 50% of the protocol duration was performed with high VO_2_, as it is known that 90% HR_max_ corresponds to >80% of VO_2max_ [29]. Mandigout et al. [22] found that training intensities greater than 80% HR_max_ for at least 25 min per session, resulted in improved VO_2max_ in children aged 10–11 years. Thus, the present circuit exercise protocol may provide an appropriate stimulus to improve aerobic fitness only in terms of intensity (>80% HR_max_) and not in terms of duration (i.e., a total of 9.2 min above >80% HR_max_).

Different exercise bout configurations during intermittent functional training protocols may modify the physiological strain [2]. For example, physiological responses may vary greatly by changing the duration of work and recovery periods and this has been known for many decades [2,30]. In the present study, the work and rest durations were very brief (7 s), and as a consequence, HR and most probably VO_2_, remained elevated throughout exercise, mimicking the responses during high-intensity continuous work. Longer exercise and rest durations are expected to cause a higher contribution of anaerobic glycolysis during exercises, combined with a drop in HR and VO_2_ during the recovery intervals, thus reducing the cardiorespiratory strain [7,31]. Along this line, Bendiksen et al. [32] reported that mean HR and time spent in high-intensity aerobic training zones was higher in ball games (2 sets × 15 min with 3 min rest) compared to circuit resistance training (30 s work and 45 s rest for 30 min). In another study, Faigenbaum et al. [33] examined acute cardiometabolic responses, applying 10 min medicine ball (2.3 kg) interval training comprising 2 sets with 30 s work and equal rest intervals. It was found that peak HR reached 178 ± 9 bpm and that mean HR ranged from 61.1% to 81.6%. These values are lower than the values reported in the present study, probably due to the extended work and interval duration. Indeed, longer exercise durations of high-intensity intermittent exercise (15–30 s at intensities >100% VO_2max_) are related with early exhaustion in child and adolescent athletes, and, in this case, a continuous bout of near-maximal exercise 80–90% VO_2max_ may be more effective to stimulate aerobic adaptations [21,34]. Moreover, muscle oxygenation, as measured by near-infrared resonance spectroscopy, is higher during shorter than longer duration exercise; rest intervals (24 s:36 s and 6 s:9 s, respectively) [35].

Another important finding of the present study was the rapid decrease of HR following both the functional sport-specific circuit training protocol and the shuttle run test (Figure 1, Table 2). Notably, the decrease in HR after 1 and 2 min of recovery was similar in both bouts and in the shuttle run test, suggesting that HR recovery in children is minimally affected by the characteristics of the preceding exercise bout. Previous studies have reported that post-exercise heart rate recovery is faster in children compared with adults, probably due to their lower work rate and less anaerobic metabolism contribution [36]. In a study comparing heart rate recovery between prepubertal, pubertal and adult males, after repeated high-intensity cycling sprints, it was shown that HR 1 min after exercise recovered by 50 ± 1 bpm, 37 ± 1 bpm and 39 ± 1 bpm, for the three age groups respectively, with no significant difference between adolescents and adults [37]. The data for HR recovery in children in that study [37] are similar with the findings of the present study (Table 2), demonstrating the rapid HR recovery in female gymnasts following this high-intensity circuit training routine. Possible reasons for the faster HR recovery may be a lower glycolytic energy supply coupled with a higher aerobic contribution and phosphocreatine resynthesis between bouts, as well as a greater parasympathetic reactivation [8,38,39].

In summary, this study presented novel and practically significant findings related to high-intensity sport-specific circuit training in child female gymnasts. However, there are certain limitations that should be acknowledged. Despite the fact that HR was continuously measured in the present study, VO_2_ responses were not evaluated. The 20 m shuttle run test, commonly used in youth athletes, is not a sport-specific test for cardiorespiratory fitness in young gymnasts. However, there is currently no other sport-specific test to asses this fitness parameter in this population. Finally, blood lactate measurements would have been informative regarding the strain placed on anaerobic glycolysis during this high-intensity workout applied in young female gymnasts. Nevertheless, it was shown that this exercise program, that is commonly applied to enhance neuromuscular performance in young female gymnasts, is characterized by an increased heart rate, above an intensity that may induce aerobic adaptations (80% HR_max_), albeit for a relatively short time. The time spent at high HR may be an appropriate stimulus for improvements in aerobic fitness in youth athletes. At the same time, performing different types of exercises from hanging and support on gymnastics apparatuses using body weight, may simultaneously enhance physical fitness and improve motor skills, especially in very young athletes. Further research should investigate the long-term effects of this training modality using different exercise durations on aerobic fitness, strength and power in child athletes of sports demanding high power and fast recovery abilities.

## Figures and Tables

**Table 1:** High-intensity circuit training program executed 5 times.

Exercise Description	Number of Repetitions/Duration
1. From hanging on high bar leg raise in tuck position to dislocate in eagle grip (L-grip), release and land on the floor	3 reps/7 s
2. From cross support facing the end of a low beam (20 cm), lateral jumps across the length of the beam	4 reps/7 s
3. Forward roll, jump with half turn (180°), backward roll and jump with half turn (180°) (without pause or extra steps)	3 reps/7 s
4. From front support on parallel bars, forward swing to straddle position and straddle travel across the length of the parallel bars	3 reps/7 s
5. From front support on low bar cast backward to horizontal	3 reps/7 s

**Table 2:** Comparison of heart rate responses during the first and the second set (set 1 and set 2) of the high-intensity circuit exercise training session.

Header	Set 1	Set 2	*p* Value	Cohen’s d
Peak heart rate (bpm)	192 ± 7	196 ± 8	<0.001	0.55
Mean heart rate (bpm)	171 ± 8	186 ± 6	<0.001	2.19
Time spent >80% ^1^ HR_max_ (min)	4.11 ± 1.19	5.09 ± 0.36	<0.001	1.15
Time spent >90% ^1^ HR_max_ (min)	2.01 ± 1.16	3.36 ± 1.65	<0.001	0.98
1 min heart rate recovery (bpm)	54 ± 13	54 ± 12	0.918	0.00
2 min heart rate recovery (bpm)	72 ± 13	69 ± 13	0.273	0.24

^1^ HR_max_: maximum heart rate attained during the shuttle run test.
